# The integrated motivational–volitional model of suicidal behaviour

**DOI:** 10.1098/rstb.2017.0268

**Published:** 2018-07-16

**Authors:** Rory C. O'Connor, Olivia J. Kirtley

**Affiliations:** 1 Suicidal Behaviour Research Laboratory, Institute of Health & Wellbeing, University of Glasgow, Gartnavel Royal Hospital, Glasgow G12 0XH, UK; 2 Center for Contextual Psychiatry, Department of Neuroscience, KU Leuven, 3000 Leuven, Belgium

**Keywords:** suicide, theory, psychology, evolutionary, risk factors

## Abstract

Suicide is a major public health concern accounting for 800 000 deaths globally each year. Although there have been many advances in understanding suicide risk in recent decades, our ability to predict suicide is no better now than it was 50 years ago. There are many potential explanations for this lack of progress, but the absence, until recently, of comprehensive theoretical models that predict the emergence of suicidal ideation distinct from the transition between suicidal ideation and suicide attempts/suicide is key to this lack of progress. The current article presents the integrated motivational–volitional (IMV) model of suicidal behaviour, one such theoretical model. We propose that defeat and entrapment drive the emergence of suicidal ideation and that a group of factors, entitled volitional moderators (VMs), govern the transition from suicidal ideation to suicidal behaviour. According to the IMV model, VMs include access to the means of suicide, exposure to suicidal behaviour, capability for suicide (fearlessness about death and increased physical pain tolerance), planning, impulsivity, mental imagery and past suicidal behaviour. In this article, we describe the theoretical origins of the IMV model, the key premises underpinning the model, empirical tests of the model and future research directions.

This article is part of the theme issue ‘Evolutionary thanatology: impacts of the dead on the living in humans and other animals'.

## 1. Introduction

Suicide is a major public health concern with at least 800 000 people dying by suicide each year across the globe and at least 20 times that number attempting suicide [1]. The pathways to suicide are complex, with suicide being the end product of an interplay of biological, clinical, psychological, social, cultural risk and protective factors [2–4]. Although knowledge of risk factors for suicide has grown markedly in recent decades [4], our ability to predict suicide is no better now than it was 50 years ago [5]. There are many reasons why the field of suicide research has not enhanced its predictive ability; key candidates include the low base rate of suicidal behaviour, as well as the fact that risk factors are often assessed in isolation and in a static rather than in a dynamic fashion [5]. In addition, until relatively, recently, there was a paucity of comprehensive theoretical frameworks that have attempted to understand the emergence of suicidal ideation and the transition from thinking about suicide to attempting suicide/dying by suicide [6].

**Figure 1.**
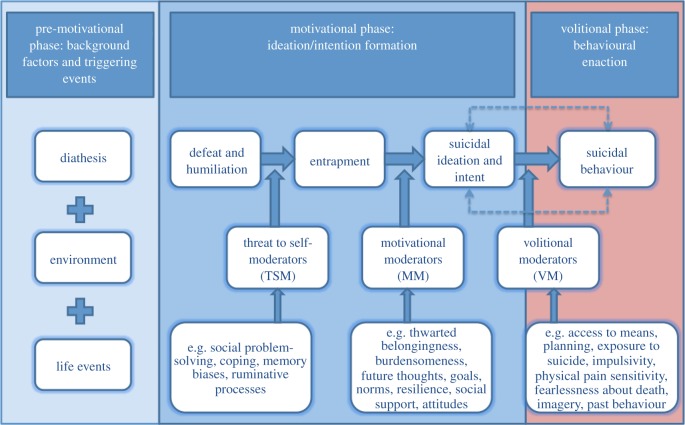
The IMV model of suicidal behaviour.

In the present paper, we focus on one such predominant framework, the integrated motivational–volitional (IMV, [6]) model of suicidal behaviour; we describe its theoretical origins, the key premises underpinning the model, empirical tests of the model and future research directions. In brief, the IMV model is a tri-partite model that describes the biopsychosocial context in which suicidal ideation and behaviour may emerge (pre-motivational phase), the factors that lead to the emergence of suicidal ideation (motivational phase) and the factors that govern the transition from suicidal ideation to suicide attempts/death by suicide (volitional phase). This is the most detailed specification of the model to date, which includes some refinements since its original exposition in 2011 (figure 1).

## 2. Theoretical origins and conceptual rationale

The guiding principle that led to the development of the IMV model was the desire to synthesize the extant evidence into a detailed theoretical framework that could make predictions about the factors that lead people to think about suicide and those factors which govern whether people act on their thoughts, i.e. attempt suicide/die by suicide. Until Joiner proposed his interpersonal theory of suicide (IPT) [7], for the most part, the theoretical literature [8–11] did not account for the distinction between the prediction of ideation versus enaction. In this regard, the IMV model is a second-generation model, which, alongside the IPT [7,12] and the three-step theory of suicide (3ST) [13], is a theoretical perspective which explains the suicidal process consistent with the ideation-to-action framework [14]. These more recent models specifically hypothesize that the factors leading to the development of suicidal thinking are distinct from those that govern behavioural enaction, i.e. attempting or dying by suicide.

As detailed elsewhere [6,15], four distinct theoretical perspectives were particularly important in the IMV model's development [9,11,16,17]. First, the backdrop to the IMV model is the diathesis–stress model [9], which recognizes that individual vulnerabilities confer elevated risk for developing suicidal ideation when activated by the presence of stressors. Examples of these vulnerabilities are personality characteristics, such as high socially prescribed perfectionism, or socio-environmental factors, e.g. socio-economic deprivation [4,18]. Combined with acute or chronic life stressors, these vulnerability factors increase the likelihood that an individual will experience an adverse psychological reaction to stress. This forms the basis of the pre-motivational phase of the IMV model, which includes background vulnerability factors.

Second, the theory of planned behaviour (TPB) [16] influenced the development of the IMV model as it contends that the strongest immediate predictor of behaviour is an individual's intention or motivation to carry out the behaviour. Crucially, the TPB delineates distinct phases of intention formation and behavioural engagement (enactment).

Central to the motivational phase of the IMV model is the relationship between defeat and humiliation, and entrapment, leading to suicidal ideation; key variables within Williams' cry of pain theory of suicide [11]. These elements are drawn from a concept known as ‘arrested flight’, which was adopted from evolutionary psychology and originally used to explain behavioural states observed in individuals with depression. Arrested flight describes the experience of feeling as though one has been brought down (defeated) and has no prospect of escape or rescue (entrapment) [19]. These concepts characterize well the ‘tunnel vision’ often observed in individuals experiencing suicidal distress, whereby suicide becomes the only perceived escape route. Humiliation also features within the cry of pain theory, but has received little substantive attention relative to defeat and entrapment.

The final theoretical perspective drawn upon within the IMV model is the differential activation hypothesis [20,21], which posits that when an individual experiences distress, an association is formed between the feeling of distress and, in this case, suicidal ideation. With each subsequent episode of distress, the pathway from distress to suicidal cognitions becomes more established and, therefore, more easily activated; negative mood also potentiates a bias towards negative information, termed ‘cognitive reactivity’ [22]. Even once an individual is no longer acutely distressed, these pathways lie dormant until triggered by a negative mood state or stress.

## 3. Key premises underpinning the motivational–volitional model of suicidal behaviour

The IMV model is a three-phase biopsychosocial framework (figure 1 and table 1) that delineates the final common pathway to suicidal ideation and behaviour. As noted above, the pre-motivational phase describes the biopsychosocial context, identifying vulnerability factors and triggering negative events. The motivational and volitional phases are operationalized at two different levels. From a higher-order perspective, the core constructs of defeat/humiliation, entrapment, suicidal ideation and suicidal behaviour form the backbone of the model and span both phases. These core constructs have the potential to be influenced by lower order moderators, with the latter defined as factors that facilitate or impede the transition within a phase (threat to self and motivational-phase moderators) or across the phases of the model (volitional-phase moderators). The key premises of the model are summarized in table 1.

**Table 1.** Key premises of the IMV model of suicidal behaviour.

	premise
1	Vulnerability factors combined with stressful life events (including early life adversity) provide the backdrop for the development of suicidal ideation.
2	The presence of pre-motivational vulnerability factors (e.g. socially prescribed perfectionism) increases the sensitivity to signals of defeat.
3	Defeat/humiliation and entrapment are the key drivers for the emergence of suicidal ideation.
4	Entrapment is the bridge between defeat and suicidal ideation.
5	Volitional-phase factors govern the transition from ideation/intent to suicidal behaviour.
6	Individuals with a suicide attempt or self-harm history will exhibit higher levels of motivational and volitional-phase variables than those without a history.
7	Distress is higher in those who engage in repeated suicidal behaviour and over time, and intention is translated into behaviour with increasing rapidity.

### (a) The pre-motivational phase: background factors and triggering events

The pre-motivational phase is comprised of a diathesis–environment–life events triad [2–4]. Diatheses take the form of biological, genetic or cognitive vulnerability factors or individual differences characteristics that increase risk of suicide. For example, decreased serotonergic neurotransmission is one such vulnerability factor for suicidal behaviour [23]. Socially prescribed perfectionism, defined as unrealistically high expectations that we believe significant others have of us [24], is another individual difference vulnerability factor that has been consistently associated with suicide risk [25,26]. According to the IMV model, socially prescribed perfectionism is hypothesized to increase the likelihood that an individual feels defeated when an interpersonal crisis occurs (heightened sensitivity to negative signals in the environment). Indeed, higher levels of perfectionism are also associated with sensitivity to emotional pain [27], another factor within the pre-motivational phase.

Understanding the social and environmental context of suicide risk has a long history [28]. More recent evidence highlights the socio-economic inequality of suicide [18] and the impact of rapid societal changes, such as economic recessions [29]. Early life adversity is also an unequivocal suicide risk factor, with evidence that it is associated with epigenetic changes in genes, cortisol (dys)regulation as well as with the (disrupted) formation of attachment relationships [2,30]. However, negative life events experienced at any stage in life confer risk [31,32].

The overarching premise of the IMV model is that the pre-motivational factors have their effect on suicide risk through their influence on the constructs within the motivational and volitional phases.

### (b) The motivational phase: emergence of suicidal ideation

Consistent with Williams' cry of pain hypothesis [11], in this phase, we focus on the psychological processes that lead to the emergence of suicidal ideation and intent. Although we acknowledge that suicidal ideation and intent are blurred but, arguably distinct constructs, at this stage there is insufficient evidence to specify whether it is useful to add another phase, which explains the movement from ideation to intent. In essence, we posit that appraisals of defeat and/or humiliation from which there is no perceived escape—a sense of entrapment—are the proximal predictors of suicidal ideation. As introduced above, sensitivity to signals of defeat may be affected by a range of factors, including socially prescribed perfectionism, pessimism and negative affect. Entrapment can be internal or external in nature; the former is concerned with being trapped by pain triggered by internal thoughts and feelings, whereas external entrapment relates to the motivation to escape from events or experiences in the outside world [19]. Feelings of entrapment are likely to give rise to agitation. Entrapment is distinct from hopelessness which is a pervasive sense of pessimism for the future [33].

The emergence of suicidal ideation is the outcome of a process beginning with feelings of defeat and humiliation. Defeat or humiliation may also be characterized by social rejection and loss, two frequently reported precipitants of suicidal distress [2,34–36]. However, entrapment is not an inevitable consequence of feeling defeated or humiliated. According to the IMV model, the presence or absence of threat to self-moderators (TSMs) renders it more or less likely that defeat leads to entrapment.

Given their established relationships with suicidal ideation and behaviour, social problem-solving [37–39], autobiographical memory biases [39–41] and rumination [42,43] are included here as TSMs. Although these factors are likely to affect entrapment as well as defeat and humiliation, we hypothesize that they will have their strongest effect on the defeat–entrapment relationship because they are implicated in problem resolution. As brooding rumination [44] is more strongly associated with suicide risk than reflection [42,43], we hypothesized that brooding would be an important moderator of the defeat–entrapment relationship. Despite limited research into the relationship between coping and suicide risk [45], given the conceptual overlap with social problem-solving, we proposed coping to be a TSM; but depending on how it is operationalized, it is likely to also moderate the entrapment–suicidal ideation relationship [45].

The final part of the motivational phase is the transition from entrapment to suicidal ideation. We posit that the presence of motivational moderators (MMs) will increase or decrease the likelihood that entrapment is translated into suicidal ideation. The MMs include factors that, when present and protective, allow the trapped individual to see alternatives, a more positive future and less pain. Reasons for living [46], attainable positive future thinking [47,48], adaptive goal pursuit [49], belongingness [12] or connectedness [50] are MMs as they are thought to buffer against the emergence of suicidal ideation and intent. Conversely, feeling a burden [51], having little or no social support [52] and depleted resilience [53] will each increase the likelihood that entrapment will be translated into suicidal ideation/intent. Consistent with the TPB, the IMV model also hypothesizes that individuals with less negative attitudes towards suicide/death are also more likely to consider suicide as an option when they are trapped [16,54]. As all human behaviour is influenced by reflective and automatic processes [55], the prediction of suicidal behaviour is no different; therefore, these attitudes are implicit as well as explicit [56,57].

### (c) The volitional phase: from suicidal ideation to suicide attempts/suicide

**Figure 2.**
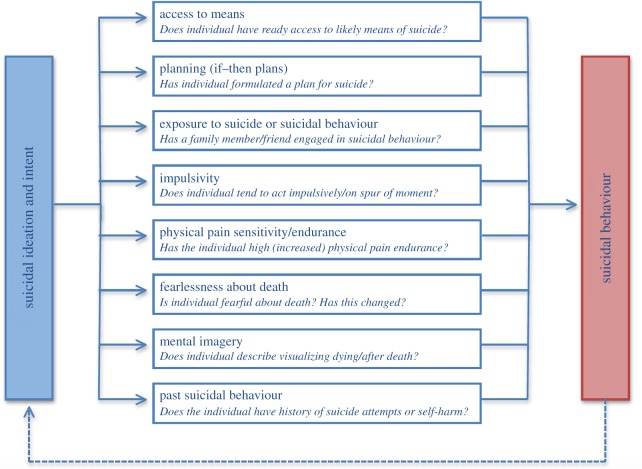
From suicidal ideation to suicidal behaviour within the IMV model: the VMs.

The final phase of the IMV model outlines the factors, entitled volitional moderators (VMs), that govern the transition from suicidal ideation/intent to enaction (the VMs are expanded upon in figure 2). Although factors such as entrapment may be associated with suicide attempts (largely due to entrapment's association with suicidal ideation), a central tenet of the IMV model is that VMs are vital for transition. Drawing from Joiner's IPT, the IMV model proposes that the components of the acquired capability for suicide (fearlessness about death and increased physical pain tolerance [12,51]) are VMs. We believe, however, that the factors that govern the transition from ideation to attempts are broader than capability. We posit that VMs can be environmental, psychological, social or physiological in nature.

Having access to the means of suicide, an environmental VM, is an important risk factor for suicide [3,58]. Exposure to the suicidal behaviour of others (family or friends) is a social VM with an established relationship with suicide risk [59,60]. There are a number of potential mechanisms that explain this relationship. For example, the suicidal behaviour of others may increase the likelihood that an individual models or imitates a loved one's suicidal behaviour. Exposure may also increase the salience and cognitive accessibility of suicide such that an individual is more likely to attempt suicide when they encounter stressors. Similarly, we hypothesize that exposure to inappropriate representations of suicide (e.g. glamorizing suicide) via traditional and new media channels may increase the likelihood that a vulnerable individual engages in a suicidal act (cf. suicidal contagion and suicide clusters, [61]).

Although there is some debate about how best to operationalize impulsivity, and the extent to which impulsivity is associated with the individual versus the act, its relationship with suicidal behaviour is evident [2,62]. The model also predicts that those with detailed (if-then) plans for their suicide or suicide attempt are more likely to attempt suicide/die by suicide than those without plans. There is also growing interest in the role of mental imagery of suicide and suicidal ‘flash forwards’ where an individual has a mental image of being dead or dying [63]. We hypothesize that mental imagery increases the likelihood of enactment as it acts as a form of cognitive rehearsal for the behaviour.

A past history of self-harm or suicide attempts is a VM. If an individual engages in suicidal behaviour once, they are statistically more likely to do so again [3]. The dotted lines in figures 1 and 2 reflect the dynamic and (for some) cyclical relationship between suicidal ideation and repeat suicide attempts. In addition, when at-risk individuals perceive themselves to have complete control over their suicidal behaviour, which may manifest itself as high capability, suicidal behaviour may be triggered directly, ostensibly bypassing the ideation/intention formation stage of the model. Although the model was developed originally to understand suicidal behaviour *per se*, the basic premises of the model also apply to self-harm, irrespective of motive. For example, volitional-phase moderators have been shown to distinguish between adolescents who have thought about self-harm and those who have self-harmed (for a wide variety of motives) [64].

## 4. Empirical tests of the model and its components

A growing number of studies have tested the IMV model or its components. As noted above, research has been supportive of the utility of VMs for differentiating between adolescents with self-harm ideation and those who enact the behaviour [64]. In another study of college students, those who reported suicidal ideation did not differ in motivational-phase variables from individuals who had attempted suicide in multivariate analyses, but they did differ on volitional-phase variables, as per the IMV model [65]. A recent study from a population-based birth cohort of 4772 adolescents also found that exposure to the self-harm of others (alongside psychiatric disorder) was the factor that most clearly differentiated those who had attempted suicide from those who had thought about suicide without making an attempt [66].

Prospective research has examined two of the central components from the motivational phase, defeat and entrapment, finding in one study that entrapment and past suicide attempts were the only multivariate predictors of readmission to hospital for self-harm at 4-year follow-up, even when controlling for depressive symptoms and hopelessness [67]. More recently, Owen *et al.* [68] found that defeat predicted suicidal ideation via entrapment at four-month follow-up in a sample of individuals with bipolar disorder. Wetherall *et al*. [69] also found that entrapment was a mediator of the relationship between defeat and suicidal ideation cross-sectionally, supporting the IMV model's prediction. Furthermore, when entrapment was high, resilience also moderated the relationship between defeat and suicidal ideation.

Entrapment is also directly related to suicidal ideation in adolescents, but it also acts as a mediator of the relationship (along with psychosomatic symptoms, resilience and depression) between anger suppression and suicidal ideation [70]. Elevated defeat, entrapment and suicidal behaviour have also been found in individuals with trauma and a diagnosis of post-traumatic stress disorder (PTSD), relative to those with trauma but no PTSD diagnosis [71]. Furthermore, defeat and entrapment mediate the relationship between PTSD symptoms and suicidal behaviour [72]. The centrality of entrapment within the suicidal process was also evident in a study of 200 adult psychiatric patients who had been hospitalized following a suicide attempt or suicidal ideation. The authors found that entrapment fully mediated the relationship between ruminative flooding, panic-dissociation and fear of dying with suicidal ideation [73].

Within the IMV model, pre-motivational factors such as socially prescribed perfectionism are posited to lead to the development of feelings of defeat, and in Wetherall *et al*.'s study [69], the relationship between socially prescribed perfectionism and defeat was partially mediated by negative social comparisons. This perception of being of a lower social rank and of making unfavourable comparisons between oneself and others is proposed to be associated with feelings of defeat and entrapment subsequently. The IMV model contends that individuals who are more sensitive to the (perceived) social evaluation of others are more likely to experience feelings of defeat and entrapment, and Wetherall *et al*.'s study provides support for this.

There have, however, been some inconsistencies in the findings between studies of defeat, entrapment and suicidal ideation. Tucker *et al*. [74] found that, in a sample of American college students, defeat was directly associated with suicidal ideation, but not indirectly via entrapment. While this is not consistent with the IMV model, central to this prediction is the temporal context of the transition from defeat/humiliation to entrapment, such that defeat is expected to temporally precede feelings of entrapment. Here defeat and entrapment were measured contemporaneously [74], which may have impacted upon whether the relationship was observed. However, also in Tucker *et al*.’s study [74], as predicted by the IMV model, the relationship between defeat and entrapment was moderated by the presence of brooding rumination, supporting rumination as a threat to self-moderator, affecting the pathway from defeat to entrapment.

Another recent study found that the rumination–suicidal ideation relationship was mediated by entrapment, but the reverse relationship whereby rumination mediated the pathway between entrapment and suicidal ideation was not significant, thus consistent with the sequential relationships outlined within the IMV model [75]. Additionally, a prospective study found baseline defeat, but not entrapment, predicted suicidal ideation at 12-month follow-up [76]. The same finding was also reported in a cross-sectional study of prisoners [77]. These findings may be due to low power, or because defeat and entrapment differ in their longitudinal relationship to suicidal ideation or the assessment of entrapment in prisoners requires closer inspection. For detailed discussion of the role of defeat and entrapment in suicide risk, see O'Connor & Portzky [78] and two recent reviews [79,80].

Novel research using an online community sample also found some support for the IMV model. On the one hand, the authors found that entrapment (alongside burdensomeness) predicted suicidal ideation cross-sectionally [81]. However, they did not find support for the moderating role of thwarted belongingness and burdensomeness in the entrapment–suicidal ideation relationship. This may simply reflect the way in which this relationship was tested and how the variables were operationalized. Drawing from the IPT [12], the IMV model proposes that it is the interaction between thwarted belongingness and burdensomeness that acts as a moderator of the entrapment–suicidal ideation relationship, as opposed to either of these variables independently. Here belongingness and burdensomeness were tested separately as potential moderators [81]. In addition, the measure of suicidal ideation encapsulates a broad spectrum of suicide-related constructs including ideation, planning and impulsivity. How we assess suicidal ideation, in itself, may introduce unwanted variability, rendering it more difficult to investigate *a priori* hypotheses.

In a new approach to understanding the relationship between risk factors, variables from the widely used Beck Scale for Suicide Ideation (SSI; [82]), which span the motivational and volitional phases of the IMV model, have been examined using network analysis in a sample of individuals who presented to hospital following a suicide attempt [83]. Results demonstrated that suicidal behaviour was more directly associated with volitional-phase variables, such as control over action and active planning, whereas factors such as reasons for living and wish to live (motivational phase factors) were more distal predictors. While innovative, this particular analysis was limited by focus on variables from the SSI, which was not designed to assess IMV model components. Future network analyses should assess all of the IMV model factors together.

A few studies have also examined the IMV model in non-Western settings. For example, Hye-Ji & Sung-Woo [84], in a sample of South Korean college students, found that entrapment mediated the relationship between defeat and suicidal ideation, as predicted by the IMV model. In sub-Saharan Africa, Atilola & Ayinde [85] applied the IMV model to examine the suicide of Sàngó, a well-known figure in the culture of the Yorùbá people, discussing how aspects from the narratives of his death map on to the IMV model. These studies provide some early evidence that the IMV model has utility for explaining suicidal behaviour in non-Western cultural settings, but this should be explored further.

A number of studies have also indirectly tested components of the IMV model. For example, innovative work with adolescents using the Card Sort Task for Self-harm (CaTS) by Townsend *et al*. [86], found that individuals outlined a process whereby negative life stressors acted as a backdrop to their distress (pre-motivational phase), leading to negative feelings and ideation about self-harm (motivational phase). Enacting self-harm behaviour was ultimately preceded by feelings of impulsivity and having the access to means for harming oneself (volitional phase). Townsend *et al*.'s work [86] supports the idea of a strong temporal component to the proposed pathways within the IMV model. In addition, work by Littlewood *et al*. [87] also found an indirect relationship between nightmares and suicidal behaviour via defeat and entrapment, supporting the idea that the combination of defeat and entrapment is particularly deleterious and leads to more severe suicidal ideation.

## 5. Key directions for future research

The shift to ideation-to-action models of suicide represents vital progress in the way we conceptualize, research, and intervene to prevent suicidal behaviour. There is still much we have yet to accomplish, however, and here we discuss a number of key opportunities and challenges for the IMV model and also suicide research more generally. As is the case for the 3ST [13] and IPT models of suicide [12], the IMV model presents a linear picture of the suicidal process, from ideation and intention formation to enactment of suicidal behaviour. Although it is important to note that the potential cyclical nature of the suicidal ideation–attempts–ideation relationship is now acknowledged within the IMV model (see dotted lines in figures 1 and 2). Nonetheless, the linear model structure does not necessarily account for repeat suicidal behaviour; as noted above, if an individual has already made a suicide attempt, it is unlikely that the process of ideation and intention formation for a repeat suicide attempt will begin anew and manifest in the same way as for a first episode of suicidal behaviour. We expect individuals who have engaged in repeated suicidal behaviours to exhibit higher levels of distress than individuals with a single episode of suicidal behaviour, and as such we expect to see higher levels of motivational and volitional-phase variables among individuals repeating suicidal behaviour. Consistent with the differential activation hypothesis [22,88], we would expect that the process between ideation and enactment shortens with repeated engagement in suicidal behaviour, such that over time the transition between intention and behaviour becomes increasingly rapid.

Given the complexity of the pathways to suicide, the model in its current form does not address the issue of whether or not particular combinations of variables from across the three phases of the model result in higher risk trajectories for suicidal behaviour. Identifying such ‘risk trajectories’ may represent important steps in generating more individually specific profiles or sub-types that may also aid our development of tailored interventions for particular groups.

As is evident from emerging literature on variability in suicidal ideation [89], context and temporal fluctuations are pivotal to our understanding of the specific circumstances under which suicidal ideation and behaviour may occur. To understand the role of context in suicidal behaviour, traditional, retrospective self-report or laboratory measures are insufficient, being highly vulnerable to recall bias and lacking ecological validity [90]. The only way to truly capture such short-term variations in risk factors is to measure these at a momentary level using techniques such as ecological momentary assessment (EMA) methods, allowing data to be collected virtually in real-time, as participants go about their daily lives [91]. Despite its clear potential, however, EMA remains an underused methodology within suicide research [56,92,93] and requires rigorous evaluation.

Since the IMV model was proposed in 2011, much progress has been made in empirically testing the model's predictions but much remains to be done. First, consistent with suicide research more generally, there is a dearth of prospective studies. The issue of temporality returns when considering the proposed temporal pathway from defeat and humiliation to entrapment, then progressing onwards to suicidal ideation. Extant research examining these constructs within the context of the IMV model has consistently investigated these variables contemporaneously [81]. The concepts of defeat and entrapment may also exhibit further nuance, potentially having both state and trait components [94]. Stability of these constructs over time has received little to no attention within the field of suicide research.

As well as new technological developments, the emergence of statistical techniques such as network analysis (e.g. [95]) provides new opportunities for addressing some of the key questions and challenges outlined above. By allowing us to compare the relative importance (centrality) of key variables associated with suicidal ideation and enactment, as well as the strength of these relationships, network analysis gives us new possibilities to investigate variations in risk trajectories in different populations. Other new methods, such as curtailment techniques, allow us to optimize the efficiency of the measures we use to assess suicidal ideation and behaviour, without compromising on their accuracy [96].

Recent advances in machine learning techniques allow the computation of optimized risk algorithms, from hundreds of different individual variable pathways, to suicidal thoughts and behaviours [97,98]. The vast majority of tools to assess the likelihood of repeat engagement in suicidal behaviour rely on self-report. A burgeoning line of research investigates possibilities for detecting cognitive reactivity towards suicide-relevant content that is outside of individuals' conscious awareness, including implicit attitudes via the Death/Life Implicit Attitudes Test [56,57,99]. Other approaches, such as the death evaluation Implicit Relational Assessment Procedure [99], have also found specific cognitive biases towards self-referent versus abstract death-related stimuli in individuals with current suicidal ideation. In short, given that behaviour is governed by reflective and automatic (e.g. implicit) processes [100], more suicide research needs to focus on these automatic (as well as reflective) processes.

## 6. Implications for intervention and suicide prevention

A corollary of the IMV model is that intervention and suicide prevention activities should be tailored to the phase of the model that the person is presently within. If an individual is distressed and feeling trapped but they are not suicidal, then clearly interventions that reduce the likelihood that suicidal ideation emerges could offer benefit. To this end, targeting factors within the motivational phase of the model should be highlighted. For example, given that entrapment is a potentially modifiable predictor of suicide attempts over time [67], this is an important treatment target. It would also make sense to incorporate the assessment of entrapment into routine clinical care alongside depression and suicidal ideation. The challenge, though, is that there are not yet any evidence-based treatments to reduce entrapment. Nonetheless, there are effective, evidence-based psychological interventions for the management of self-harm that can be drawn from Hawton *et al.* [101]. If an individual is actively suicidal, in addition to trying to alleviate their suicidal distress, it is vital that interventions to reduce the likelihood that they act on their thoughts are prioritized. For example, safety planning [58] is one such promising intervention which targets VMs. Another is a volitional helpsheet (VHS) [102] that encourages an individual to make if-then plans to reduce the likelihood that their suicidal thoughts trigger a suicide attempt. Recent evidence suggests that a VHS may offer promise (as an adjunct to usual care), especially among those with a past history of self-harm [102,103]. More generally though, theoretical models such as the IMV model should be a starting point for the development of interventions, because they specify the potential mechanisms that should be targeted, thereby increasing the likelihood of interventions being effective [104]. Finally, at the macro-level, suicide prevention efforts need to urgently tackle inequality, poverty and disadvantage [18,105], key drivers of suicide (pre-motivational phase).

## 7. Summary and conclusion

We have presented the IMV model, a contemporary ideation-to-action model of suicidal behaviour. The tri-partite IMV model contends that suicide is a behaviour, preceded by ideation and intention formation and, crucially, it seeks to explain the transition from suicidal ideation to behavioural enactment. Empirical support for the model is growing; however, there remain a number of challenges, as well as opportunities, to be addressed in future research; understanding the roles of temporality and complexity of variable interactions within the model is a priority.

## Data accessibility

This article has no additional data.
